# What is stirring in the reservoir? Modelling mechanisms of henipavirus circulation in fruit bat hosts

**DOI:** 10.1098/rstb.2019.0021

**Published:** 2019-08-12

**Authors:** Emma E. Glennon, Daniel J. Becker, Alison J. Peel, Romain Garnier, Richard D. Suu-Ire, Louise Gibson, David T. S. Hayman, James L. N. Wood, Andrew A. Cunningham, Raina K. Plowright, Olivier Restif

**Affiliations:** 1 Department of Veterinary Medicine, University of Cambridge, Cambridge CB3 0ES, UK; 2 Department of Microbiology and Immunology, Montana State University, Bozeman, MT 59717, USA; 3 Department of Biology, Indiana University, Bloomington, IN 47405, USA; 4 Environmental Futures Research Institute, Griffith University, Nathan, Queensland, QLD 4111, Australia; 5 Department of Biology, Georgetown University, Washington, DC 20007, USA; 6 School of Veterinary Medicine, College of Basic and Applied Sciences, University of Ghana, Legon, Accra, Ghana; 7 Institute of Zoology, Zoological Society of London, London NW1 4RY, UK; 8 Molecular Epidemiology and Public Health Laboratory, Infectious Disease Research Centre, Hopkirk Research Institute, Massey University, Palmerston North, 4442, New Zealand

**Keywords:** disease dynamics, henipavirus, *Eidolon helvum*, fruit bats, zoonosis, reservoir hosts

## Abstract

Pathogen circulation among reservoir hosts is a precondition for zoonotic spillover. Unlike the acute, high morbidity infections typical in spillover hosts, infected reservoir hosts often exhibit low morbidity and mortality. Although it has been proposed that reservoir host infections may be persistent with recurrent episodes of shedding, direct evidence is often lacking. We construct a generalized SEIR (susceptible, exposed, infectious, recovered) framework encompassing 46 sub-models representing the full range of possible transitions among those four states of infection and immunity. We then use likelihood-based methods to fit these models to nine years of longitudinal data on henipavirus serology from a captive colony of *Eidolon helvum* bats in Ghana. We find that reinfection is necessary to explain observed dynamics; that acute infectious periods may be very short (hours to days); that immunity, if present, lasts about 1–2 years; and that recurring latent infection is likely. Although quantitative inference is sensitive to assumptions about serology, qualitative predictions are robust. Our novel approach helps clarify mechanisms of viral persistence and circulation in wild bats, including estimated ranges for key parameters such as the basic reproduction number and the duration of the infectious period. Our results inform how future field-based and experimental work could differentiate the processes of viral recurrence and reinfection in reservoir hosts.

This article is part of the theme issue ‘Dynamic and integrative approaches to understanding pathogen spillover’.

## 1. Introduction

Pathogen circulation in reservoir hosts is an essential precursor to spillover but is often poorly understood relative to post-spillover processes. Bats are an especially important clade to study, as they host a uniquely rich set of viruses—more viruses per species than even rodents [1], including many important emerging zoonoses [2,3]. Bats host at least six of the World Health Organization's top ten named priority pathogens with potential to create a public health emergency [4]. The mechanisms that allow for the circulation of such otherwise virulent viruses in their reservoir hosts, however, are poorly understood despite their enormous consequences for human health.

Henipaviruses are hosted by fruit bats (family Pteropodidae) and include Hendra virus (HeV) in Australia and Nipah virus (NiV) in Asia [5–7], which are among the bat-borne pathogens considered by the WHO and others to have the highest pandemic potential. Both HeV and NiV cause almost annual outbreaks in horses and people, respectively. Human fatality rates are greater than 50% [8]. Spillover has occurred both directly from bats to people (e.g. NiV) [9–12] and indirectly via amplifying or bridging hosts, namely pigs for NiV and horses for HeV [13–15]. Henipaviruses have also been detected in fruit bats in Africa [16], and antibodies to them have occasionally been detected in people and pigs, although no human cases have yet been documented [17–19]. The nature of henipavirus circulation in the reservoir—including the possibility that these viruses can persist in individual hosts and be impacted by environmental forces—has strong implications for the risk and drivers of spillover to people [12,20,21].

The hypothesis that henipavirus infections may be recurrent (i.e. oscillating between latent and acute infection) in their bat hosts has been gaining support (reviewed in [22]). Evidence includes simultaneous viral shedding of henipaviruses from a large number of individuals in a single roost during presumed times of physiological or nutritional stress [23,24]; serological conversions of bats that had previously exhibited apparent clearance [25] (though re-exposure from an external source cannot be ruled out); ongoing henipaviral circulation in small island populations [26,27]; and long-term persistence of circulating henipaviruses in small, closed populations [28]. However, our incomplete understanding of bat immunology and the difficulty associated with isolating henipaviruses from bats have rendered it challenging to determine what these observations mean in terms of bats' immunity, clearance and transmission of these pathogens [22]. Simple models of plausible latent, recurring infection (e.g. the ‘susceptible–infected–latent–infected’, or SILI, model) have been analysed theoretically but not empirically applied to this system [22,29].

Rather than comparing alternative, arbitrary models of bat-virus dynamics, we decided to systematically explore a comprehensive set of hypotheses about the cycle of henipavirus infection and immunity in bats. We expand upon the classical compartmental SEIR (‘susceptible, exposed, infectious, recovered’) framework to cover a comprehensive range of models of infection dynamics, including features of recurrence, reinfection and non-infectious infection. We statistically fit these models to a longitudinal serological dataset of a breeding, captive colony of *Eidolon helvum* held in Ghana for nine years. In line with empirical evidence [27], we include a seasonal birth pulse, maternally derived immunity and a simple age structure in our set of models. We use the results of the cross-model comparison to predict the most likely within-host dynamic features—including cycles of recurrence and reinfection, clearance of infection, and probable parameter values—of African henipavirus infections in their bat reservoir hosts.

## 2. Material and methods

### (a) Data

Individual-level serological data were collected longitudinally from a captive colony of *E. helvum* established in Achimota forest, Accra National Zoo in Accra, Ghana as described in [28]. The colony is separated from the surrounding forest by a solid roof and two layers of wire mesh, and captive bats have been isolated from all other bats since colony establishment. After the initial capture of 77 wild *E. helvum* by January 2010, the bats have been breeding in captivity, and since 2012 the population has oscillated between approximately 100 and 120 individuals. Blood has been collected from the tagged bats 1–5 times per year since the establishment of the colony, and seroprevalence has been assessed using a Luminex assay. Antibody levels were represented using the mean fluorescence index (MFI) and the seropositivity cut-off was set at 110 MFI (electronic supplementary material, figure S1) [28,30].

### (b) The generalized SEIR model

**Figure 1.**
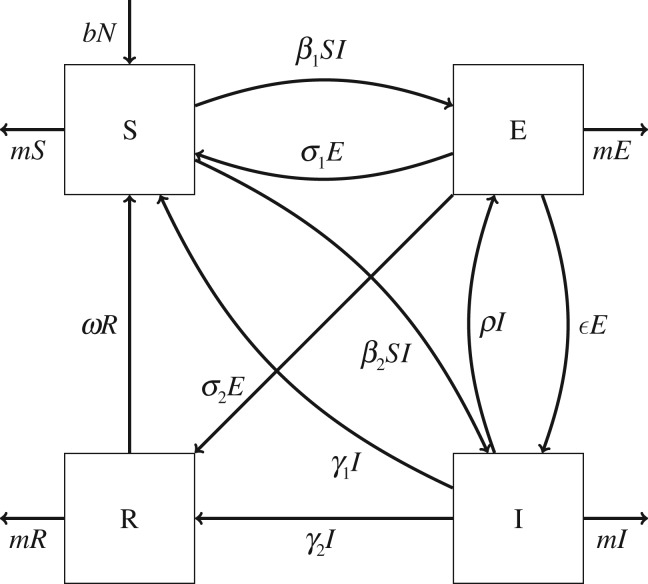
Diagram of generalized SEIR model showing all possible connections between compartments. Parameters represented include the transmission rate *β_i_* (where *i* is in {1, 2}), the recurrence rate *ε*, the ‘latency’ rate *ρ*, the immune waning rate *ω* and the clearance rates from latent and acute infection, *σ_j_* and *γ_k_* (where *j* and *k* are both in {1, 2}), respectively. While these parameters indicate the same state transitions in all submodels, their biological representations may vary; e.g. in a model with *β*_1_ > 0 and *ρ* = 0, a high value of *σ*_2_ indicates non-infectious infection rather than clearance from recurring infection.

Because within-host dynamics of henipaviruses in bats are so poorly understood, we opted to allow for multiple assumptions about the existence of immunity, heterogeneity in the form of infection and ability of infections to clear or recur. We developed a framework that generalizes the SEIR model, composed of a subset of all possible combinations of transitions among four state variables (figure 1 and table 1):

1. S: susceptible and must undergo infection to become immune;
2. E: infected but not infectious (‘exposed’)—either incubating or latently infected;
3. I: both infected and infectious, contributing to the force of infection;
4. R: recovered/immune and must lose immunity to be reinfected.

**Table 1.** Parameter names and values used in all models. The parameters *β_i_*, *σ_j_* and *γ_k_* can each occur in two forms where (*i*, *j* and *k* are each in {1, 2}), but only one of each pair is nonzero for any submodel. The birth pulse timing parameter *ϕ* corresponded to a birth pulse peak occurring in April in Accra, Ghana [28]. The *R*_0_ range included subcritical values owing to the small population of the captive colony.

symbol	parameter meaning	value constraints	source
*R*_0_	basic reproduction number	0.25+	fit for all models
*β*_1_	transmission rate to E	—	calculated from *R*_0_
*β*_2_	transmission rate to I	—	calculated from *R*_0_
*ω*	immune waning rate	0+	fit for relevant models
*ρ*	‘latency’ rate (I→E)	0+	fit for relevant models
*ε*	incubation/recurrence rate (E→I)	0+	fit for relevant models
*σ*_1_	clearance rate (→S) from E	0+	fit for relevant models
*σ*_2_	recovery rate (→R) from E	0+	fit for relevant models
*γ*_1_	clearance rate (→S) from I	0+	fit for relevant models
*γ*_2_	recovery rate (→R) from I	0+	fit for relevant models
*ϕ*	birth pulse timing	4.5	[28]
*s*	birth pulse synchronicity	14.3	[31]
*c*	birth pulse scalar	1.53	calculated to balance deaths
*m*	adult death rate	0.186 year^−1^	[32]
*m_j_*	newborn and juvenile death rate	0.796 year^−1^	[32]
*ω_m_*	maternal antibody waning rate	1.79 year^−1^	[27]
*μ*	juvenile maturation rate	2.27 year^−1^	for 1-year juvenile stage
*N*	population size	13–123	exact sample numbers
*k*	population carrying capacity	100	estimated to match observed population oscillations (100–120)

We added the additional constraints that viral transmission occurs directly, that any infection requires an incubation period for either all hosts or no hosts (i.e. for any one model all transmissions occur either to E or to I), that the model must include some way for individuals to enter an infectious (I) compartment, and that recovery from either infected state is immunizing in either all cases or none (although individuals from E may develop immunity while those from I clear infection without immunity, or *vice versa*). These constraints restrict the general framework shown on figure 1 to 46 submodels, including classical SIR, SIRS, and SEIR/SEIRS models, as well as a model identical to the SILI model previously proposed for henipavirus dynamics [22] (where L (latent infection) is the same as our E (exposure to a disease--which may include latent infection) compartment), and models including more elaborate types of recurrent infection with the possibility of either temporary or lifelong immunity.

For any model within this framework, the basic reproduction number *R*_0_ can be calculated using the next generation matrix method [33] as:

$$R_{0}=\frac{\varepsilon \beta_{1}N+\beta_{2}N(\varepsilon +\sigma_{1}+\sigma_{2}+m)}{(\varepsilon +\sigma_{1}+\sigma_{2}+m)(\gamma_{1}+\gamma_{2}+m+\rho )-\varepsilon \rho }.$$

Owing to the diversity of submodels contained within this framework, we use the following notation system to refer to each uniquely:

— Square brackets represent loops of exposed/latent and acute infection (i.e. recurrent infection); individuals can flow from the last compartment within the set of brackets to either the first compartment within the brackets or the first compartment to the right of the brackets; e.g. S[IE] is our notation for the previously developed SILI model and S[IE]R indicates recurrent infection where latently infected individuals can develop immunity.
— Parentheses indicate one of two possible routes for the preceding compartment; e.g. E(S)I indicates that exposed individuals can directly become either susceptible or acutely infected.

To allow for maternal immunity and emulate age structure, we also incorporated a simple age- and sex-stratified structure into the model. This structure included newborn (up to 6.7 months to correspond to estimates of maternal antibody waning [27]), juvenile (up to 1 year), adult male and adult female classes. Newborns and juveniles have a higher mortality rate than adults, corresponding to previous estimates [32]; newborns are born with maternally derived immunity if and only if born to an immune mother [28]. Births occur according to a yearly birth pulse as previously developed [34]. Our newborn, juvenile and adult age classes are related to dynamic characteristics and do not correspond exactly to morphologically assessed age categories [35]. Newborns in our model are instead characterized by potential maternal immunity and correspond to individuals typically labelled neonate or (young) juvenile, while our adult age classes are characterized by higher annual survival rates than juveniles and include both adult and sexually immature (i.e. subadult) individuals between approximately 1 and 2 years of age. We calculated *R*_0_ based on the adult mortality rate.

### (c) Fitting models to data

**Figure 2.**
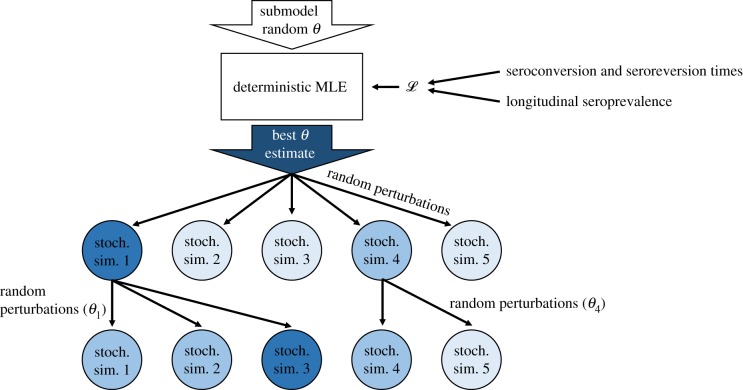
Diagram of model fitting procedure for a single submodel with five particles and two iterations. The first stage of fitting is maximum-likelihood estimation (MLE) of the deterministic version of the submodel, where the likelihood function incorporates two types of data: estimates of seroconversion and seroreversion times, and sampled seroprevalences over time. The best parameter estimate (*θ*) is perturbed slightly for each particle (circles) and then used to simulate the stochastic version of the submodel once per particle. The likelihoods of each simulation are calculated (here, darker colours represent higher likelihoods) and the parameters from the highest-likelihood particles (here, particles 1 and 4) are sampled in proportion to their likelihood-based weights. These are perturbed again and used in a new round (i.e. iteration) of sampling. (Online version in colour.)

To account for both the goodness-of-fit of model trajectories and their chances of persisting in this small, closed population, we fit models to the data in two stages (figure 2). In both stages, we used a likelihood function that accounts for overall observed seroprevalence and observed distributions of seroconversion and reversion times, with the first stage using the deterministic variant of each model. In the second stage, we fit the stochastic variant of each model to additionally account for the chance of stochastic persistence in this small, isolated population. The two stages were:

1. Maximum-likelihood optimization of the deterministic variant of the model with a burn-in time of 300 years and initial parameters sampled from a Latin hypercube sample (*n* = 100) owing to the frequency of parameter values resulting in a likelihood of zero.
2. Iterated particle filtering [36] (100 iterations on 10 000 particles with a cooling factor of 1% per iteration; see electronic supplementary material, text 2.2 for additional details). For the first iteration, we performed 10 000 stochastic simulations of the submodel with parameters perturbed from the initial parameters determined in step 1. Subsequent iterations involve:
  (a) Calculating the likelihoods of all 10 000 simulations of the previous iteration.
  (b) Sampling starting parameter sets proportional to weights calculated from those likelihoods.
  (c) Perturbing those starting parameter sets with an initial standard deviation of half the initial parameter value (with a minimum standard deviation of 0.1 for *R*_0_). This standard deviation ‘cooled’ by a factor of 0.01 per iteration.
  (d) Finally simulating exact stochastic trajectories of each model using an adaptive *τ*-leaping algorithm (by repeatedly sampling transition events for each time step *τ*; electronic supplementary material, text 2.3).

Owing to uncertainties about the mechanisms of antibody responses in bats [22,37], we performed this analysis under two different assumptions about serological status. In the first, we assumed that all non-susceptible individuals are seropositive (i.e. the E, I and R compartments). In the second, only the R compartment is seropositive. We refer to these sets of assumptions as EIR+ and R+, respectively.

The likelihood function for each of these stages was based on cross-sectional seroprevalences, the probabilities of different seroconversion/reversion pathways within each model and the expected time for an individual to traverse that pathway; we fit these components both to population-level seroprevalence at each sampling point and to the range of possible timings of all observed seroconversion/reversion events (i.e. a uniform distribution of times between the minimum and maximum possible times based on the sampling dates). Additional information on the likelihood function can be found in electronic supplementary material, text 2.1.

### (d) Model comparison

For each set of assumptions, we created a composite model by averaging parameters by Akaike weight (derived from Akaike information criteria, AIC) for that assumption [38]. We also used the Akaike weights to estimate the relative importance for each model parameter and several model features comprised of parameter and model specification combinations, such as recurrent latent infection (electronic supplementary material, text 2.4). For each set of assumptions, we calculated relative importance for each possible parameter and feature as the summed weight of all models containing the relevant parameter(s).

## 3. Results

### (a) Model comparison

**Figure 3.**
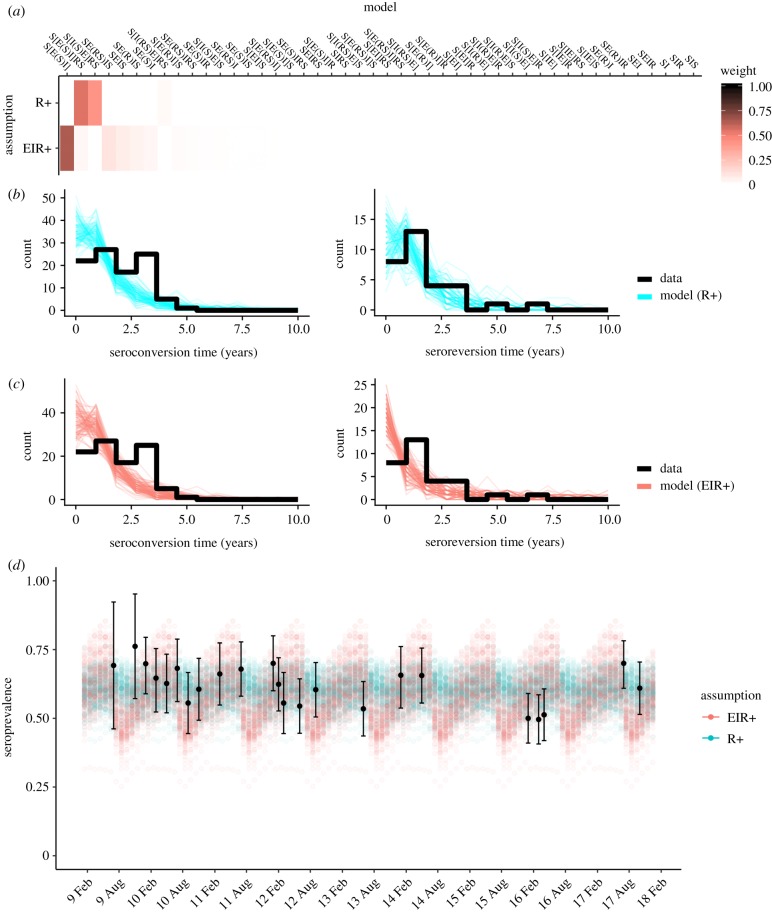
Model fits under different serological assumptions. (*a*) Akaike weights for each model and assumption. (*b*,*c*) 100 simulated sets of predicted versus observed seroconversion and seroreversion times under the R+ assumption (blue) and the EIR+ assumption (pink). (*d*) 100 stochastic simulations of the best-fitting model under each set of assumptions (EIR+ in pink; R+ in blue). Each simulation used parameters sampled according to particle weights. Measured seroprevalences and 95% binomial confidence intervals are shown in black.

Top-fitting models were able to reproduce observed patterns of seroprevalence, seasonality and distributions of seroconversion/seroreversion times (figure 3). The two sets of serological assumptions (EIR+ and R+) resulted in different top models according to AIC (figure 3*a*), although both predicted recurrent cycles of acute and latent infection and spontaneous clearance of latent infection (electronic supplementary material, figures S6 and S7):

— E, I and R seropositive (EIR+): S[E(S)I] (Akaike weight of 0.64): model with initial exposure that can either clear without acute infection or may result in acute infection. Once acute, may recur through cycles of latent infection or may clear.
— R only seropositive (R+): S[E(S)I]RS (Akaike weight of 0.55): as in EIR+ model, but acute infection may result in temporary immunity.

Many model structures were unable to adequately predict both observed serological patterns and viral persistence in the captive colony under certain serological assumptions. For example, under the EIR+ assumption, SI/SEI (i.e. lifelong infection), S[IE]R/S[EI]R (i.e. recurrent latent infection with eventual lifelong immunity) and S[EI]/S[IE] (i.e. lifelong recurrent infection) models all resulted in a likelihood of zero when applied to our longitudinal dataset. This was often, but not always, because these models cannot produce both seroconversions and seroreversions (e.g. under the EIR+ assumption, only models where infected bats can eventually return to susceptibility can produce seroreversions).

**Figure 4.**
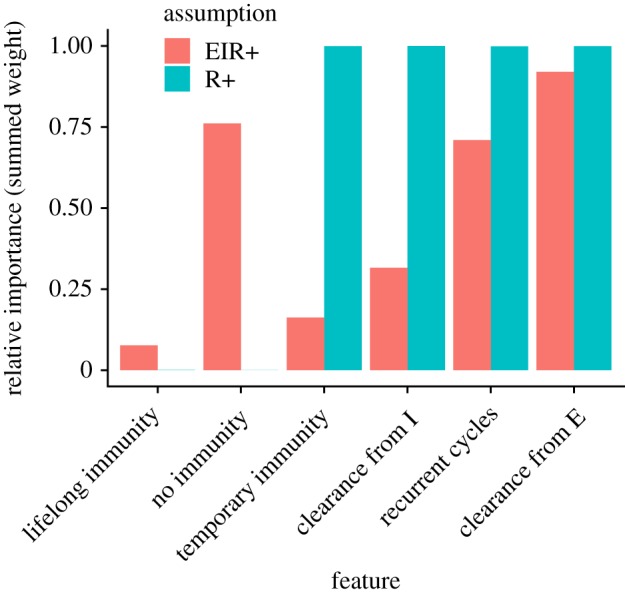
Relative feature importance (i.e. summed Akaike weights of models incorporating each feature; see electronic supplementary material, text 2.4 for feature definitions) under each set of assumptions about serological status (EIR+ and R+). (Online version in colour.)

Under the assumption that antibodies represent immunity (i.e. R+) all likely models included two types of infection cycles: recurrent latent infection and reinfection following viral clearance (figure 4). Under the EIR+ assumption, potential models were more varied, but rarely included sterilizing immunity (i.e. any R compartment) and often included potentially recurrent latent infection.

### (b) Parameter estimates

**Figure 5.**
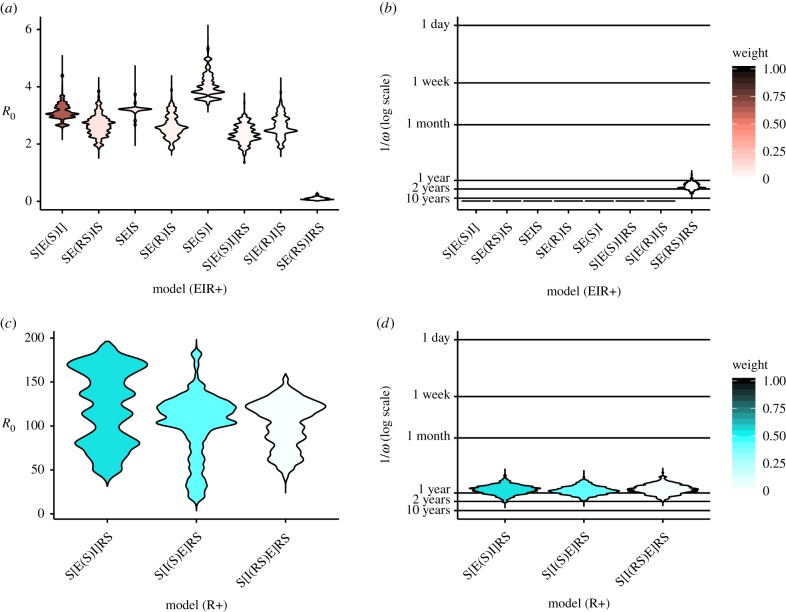
Distributions of predicted parameter values for models with at least 1% Akaike weight under the EIR+ (*a*,*b*) and R+ (*c*,*d*) assumptions. *R*_0_ values (*a*,*c*) and immune waning durations (*b*,*d*) are weighted by particle likelihood in last 10 iterations of stochastic captive colony fitting procedure. Models are ordered according to decreasing weight. Most models under the EIR+ assumption result in identical predictions of lifelong immunity (*b*) because they do not include the relevant parameter (*ω*). (Online version in colour.)

Under the EIR+ assumption, several key parameter estimates were consistent across models, especially *R*_0_ (figure 5*a*) and the immune waning rate *ω*. Most high-likelihood values of *R*_0_ fell between 2 and 4, with a composite mean of 3.0. However, the R+ assumption resulted in extremely high *R*_0_ values (up to about 200; figure 5*c*). The composite mean *R*_0_ values for this assumption was 112.3.

Estimated immune waning times were remarkably consistent across all models and both sets of assumptions (figure 5). Under the EIR+ assumption, all but one model predicted immunity lasting either 1–2 years or lasting lifelong (10+ years) or longer on average. Under the R+ assumption, predicted immunity lasts just under 1 year for all probable submodels.

While recurrence and reinfection after viral clearance were supported in nearly all high-likelihood models, the balance of these mechanisms differed by serological assumption (table 2). However, under both assumption sets seroconversion and seroreversion processes were best supported by frequent cycles of recurrent infection and occasional clearance.

**Table 2.** Top and weighted composite models under each set of serological assumptions. All top and composite models include reinfection, recurrence and non-infectious infections. Composite models are mean parameters weighted by submodel Akaike weights.

	EIR+		R+	
	top model	composite	top model	composite
representation	S[E(S)I]	—	S[E(S)I]RS	—
*R*_0_	2.0	3.0	66.7	112.3
shedding duration	1.0 months	2.7 days	1.6 weeks	4.5 h
latency/incubation period	2.1 h	1.8 h	3.0 h	2.9 h
immunity duration	—	88 years	2.3 years	1.3 years

Weighted estimates for other parameters were more variable (electronic supplementary material, figures S2–S5) but exhibit several trends. For example, the duration of acute infection predicted under the R+ assumption is between hours and about one week under all but three models with nonzero likelihoods; these three models predict long infectious periods but are three of the four worst-fitting models. For both sets of assumptions, cycles of acute and latent infection are predicted to be very short (between hours and days).

## 4. Discussion

Observed patterns of seroprevalence, seroconversion, seroreversion and persistence of henipaviruses in a captive colony of *E. helvum* in Ghana were best explained by cycles of reinfection with occasional viral clearance, possibly alongside cycles of recurrent latent henipavirus infection and/or non-infectious infections. For the best-fitting model under the EIR+ assumption (i.e. individuals in the E, I and R compartments are seropositive), a latently infected bat is about 75 times more likely to undergo at least one more short bout of acute infection than to spontaneously clear infection. This leads to an expected duration of infection (including both latent and acute stages) of about 4.5 years. For the best-fitting model under the R+ assumption (i.e. only individuals in the R compartment are seropositive), an acutely infected bat is about 40 times more likely to return to a latent state than to recover and develop temporary immunity, with an expected duration of infection of about 10 months. These expected durations are, however, highly variable even for a single parameter value, because there is a wide distribution of the number of infection cycles that a single individual may experience. Minimum infection times are possible on the scale of about a day (between 1–3% of individuals under both sets of assumptions), while maximum infection times may last throughout a bat's expected lifetime (although with less than 0.1% probability). The variability in infection length and frequent support for multiple infection pathways may suggest high individual heterogeneity in response to infection; e.g. some individuals may be able to effectively suppress infections while others, perhaps in response to pregnancy or other sources of physiological stress [21,39], experience acute infection or recurrence. Measuring differences in infection and antibody dynamics at the individual level could provide additional support for the existence of multiple infection pathways and could help disentangle these processes.

Both sets of serological assumptions (EIR+ and R+) consistently predict rapid cycles of acute and latent infection that correspond to the cyclic nature of seroprevalence in the observed data. This suggests that viral shedding is sporadic, in accordance with observations of henipaviruses in nature, although we note that a transition time of a few hours is unlikely to represent a true immune response [16,23]. Our likelihood function may favour excessively short cycles of acute and latent infection because these can provide a wide range of probable serological transition times. Although experimental infection studies have failed to provide reliable data on the patterns and duration of henipavirus shedding [6], our results indicate that acute–latent infection cycles are able to reflect naturally observed variation in serological transition times (perhaps reflecting individual heterogeneity [40] or dose-dependency [41] in immune responses).

Observed patterns of seroprevalence, seroconversion and seroreversion could not be explained by models with simple immunizing infection or recurrent latent infection alone. Especially under the R+ assumption, most models had likelihoods of zero, including many models with immune waning. SEIR models—which may apply to Marburg virus dynamics in fruit bats [31,42,43]—and SEI models with or without immunizing asymptomatic infections—which may explain rabies persistence in neotropical and temperate bats [44–46]—notably could not explain observed patterns of henipavirus serology in the captive *E. helvum* colony under either serological assumption. Even models of lifelong latent infection were unable to explain these patterns under our current model assumptions. This includes the S[IE] model, which had been suggested (under the acronym of SILI) for henipavirus dynamics in fruit bats [22]. Thus, while our study supports the existence of recurrent infection in bats, it also suggests a need for additional features of the cycle of infection and immunity.

Variations both within and between assumption sets—including apparently unrealistic predictions—are informative about which dynamic features are required to explain observed patterns. The extremely high predicted *R*_0_ values under the R+ assumption, for example, may suggest that long-term viral persistence in this small, closed population is unlikely within plausible parameter ranges if all seropositive individuals are immune. Indeed, the data imply that 60–70% of bats would be immune under the R+ assumption, which may require a very high value of *R*_0_ for the virus to persist; these values allow some individuals to be infected long-term, maintaining infection in the population and avoiding stochastic extinction that is otherwise likely with only a few dozen susceptible individuals. However, owing to the lack of prior constraints on the range of parameter values explored during the fitting process, we cannot rule out that there are other plausible parameter sets with lower *R*_0_ values that were excluded by our likelihood-maximization method.

One of the limitations of our analysis is the remaining uncertainty about the interpretation of serological data in the absence of virological data. Ideally, measurements of both infection and serological status could allow stronger inferences. However, while viral shedding in urine has been readily detected in wild bat populations, no consistent, accurate and noninvasive test of an individual's true henipavirus infection status currently exists. Some immunological differences between bats and other mammals may exist; as additional research clarifies the role of their antibody responses to infection, and henipavirus infection in particular, the appropriate set of serological assumptions may become clearer [43,47–49]. In addition, our analysis relied on the classification of bats as either seropositive or seronegative, which is achieved by choosing a MFI cut-off for the Luminex serological assays. Because interpretation of bats' antibody responses to henipavirus infection remains uncertain [30], this may introduce some bias in our results. However, the distributions of seroconversion times and seroreversion times based on the data remain similar across a wide range of cut-off values (electronic supplementary material, figure S1).

Explicit modelling of antibody titres and measurement uncertainty (if necessary, with an assay that more consistently and directly maps to individual infection status) could improve inference but would require additional information about the role of antibodies in bats' response to henipaviruses. Modelling antibody titres instead of seropositive versus seronegative status would also require more frequent sampling timepoints. Other limitations of our analysis include the assumption of a steady state within the colony. More longitudinal studies of bat henipavirus dynamics in wild populations could resolve these issues, although low rates of recapture make such studies difficult [50]. Finally, any additional bounds on our parameters could improve inferences. For example, constraining the duration of acute infection/viral shedding in particular could prevent any bias that our captive colony fitting algorithm shows toward short acute-latent cycle times.

Despite these limitations, we have narrowed the range of plausible hypotheses for persistence and circulation of henipaviruses in a fruit bat reservoir host in Africa, using uniquely long-term and well-controlled data from a captive colony. Because the captive colony in this study has been isolated from wild bats, has had minimal human intervention, has a well-documented demographic history and has demonstrated ongoing henipavirus circulation for almost a decade, it is an ideal system to study the long-term individual- and population-level dynamics of henipaviruses with minimal risk of an external force of infection. Our generalized SEIR model framework has allowed us to compare a diverse range of models and parameters, representing many potential within-host mechanisms rather than assuming such mechanisms in the context of uncertain serological interpretation.

Our expanded SEIR framework lends itself to exploring hypotheses for the hidden process of viral circulation in reservoir hosts more broadly. This framework could be useful for generating transmission and within-host hypotheses for other low-morbidity pathogens. Application of this framework to such pathogens may be particularly useful to develop testable predictions to target field-based and experimental work [51]. In the case of henipaviruses in bats, models of reinfection following viral clearance and seasonally recurring latent infection have been considered as possible explanations of seasonal shedding [22]. Future work on the relationship between antibody presence and infection status, as well as examination of heterogeneity in responses to infection, could help disentangle these hypotheses further. While we cannot conclusively say which mechanism underlies this process, we have extended these explanations into a wider set of hypotheses, applied them to several types of real-world data and supported the existence of certain dynamic features of henipaviruses in *Eidolon helvum*.

## Supplementary Material

Supplementary material

## Data accessibility

Data available from the Dryad Digital Repository: https://doi.org/10.5061/dryad.5v3s28v [52].
