# Supplementary material for: What is stirring in the reservoir? Modelling mechanisms of henipavirus circulation in fruit bat hosts
Source: Philos Trans R Soc Lond B Biol Sci. 2019 Aug 12;374(1782):20190021. doi: 10.1098/rstb.2019.0021 (PMC6711305; doi:10.1098/rstb.2019.0021)
Supplement: Supplementary material [file rstb20190021supp1.pdf]

Supplementary Material: What's stirring in the reservoir? Modelling mechanisms of henipavirus circulation in fruit bat hosts

Emma E. Glennon, Daniel J. Becker, Alison J. Peel,
Romain Garnier, Richard D. Suu-Ire, Louise Gibson, David T.S. Hayman
James L. N. Wood, Andrew A. Cunningham, Raina K. Plowright, Olivier Restif

1 Cutoff for serological assay

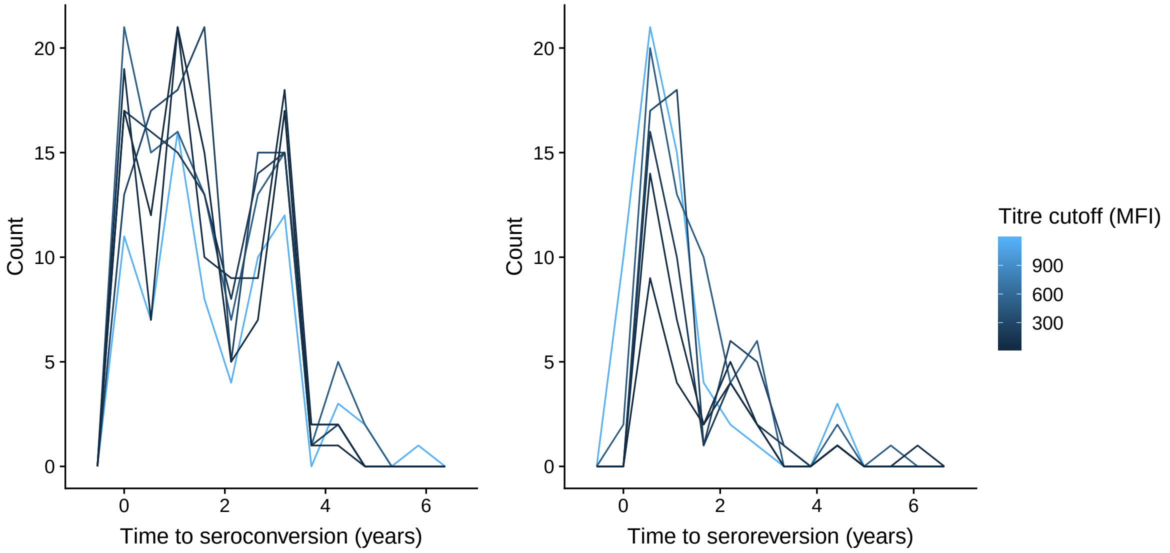

Figure 1: Distributions of observed seroconversion and seroreversion times by MFI cutoff used to differentiate seropositive and seronegative individuals by Luminex assay.

2 Additional methods

We added a simple age structure into our SEIR model, including some individuals born with maternal immunity (if born to immune mothers). The (deterministic version of the) full model including age structure is represented by the following differential equations, where parameters are used as in Table 1 in the main text and M, F, J, and N represent adult male, adult female, juvenile, and newborn individuals, respectively (although the Ma compartment itself represents maternally immune newborns):

$$
\begin{aligned}
b(t) &= ce^{-s\cos^2(\pi t-\phi)}\\
\frac{dS_F}{dt} &= \mu\frac{S_J}{2} - (m\frac{N}{k} + (\beta_1+\beta_2)(I_F+I_M+I_N+I_J))S_F + \sigma_1 E_F + \gamma_2 I_F + \omega_2 R_F\\
\frac{dE_F}{dt} &= \mu\frac{E_J}{2} - (m\frac{N}{k} + \sigma_1+\sigma_2+\epsilon)E_F + \beta_1(I_F+I_M+I_N+I_J))S_F + \rho I_F\\
\frac{dI_F}{dt} &= \mu\frac{I_J}{2} - (m\frac{N}{k} + \gamma_1+\gamma_2+\rho)I_F + \beta_2(I_F+I_M+I_N+I_J))S_F + \epsilon E_F\\
\frac{dR_F}{dt} &= \mu\frac{R_J}{2} - (m\frac{N}{k} + \omega)R_F + \sigma_2 E_F + \gamma_2 I_F\\
\frac{dS_M}{dt} &= \mu\frac{S_J}{2} - (m\frac{N}{k} + (\beta_1+\beta_2)(I_F+I_M+I_N+I_J))S_M + \sigma_1 E_M + \gamma_2 I_M + \omega_2 R_M\\
\frac{dE_M}{dt} &= \mu\frac{E_J}{2} - (m\frac{N}{k} + \sigma_1+\sigma_2+\epsilon)E_M + \beta_1(I_F+I_M+I_N+I_J))S_M + \rho I_M\\
\frac{dI_M}{dt} &= \mu\frac{I_J}{2} - (m\frac{N}{k} + \gamma_1+\gamma_2+\rho)I_M + \beta_2(I_F+I_M+I_N+I_J))S_M + \epsilon E_M\\
\frac{dR_M}{dt} &= \mu\frac{R_J}{2} - (m\frac{N}{k} + \omega)R_M + \sigma_2 E_M + \gamma_2 I_M\\
\frac{dMa}{dt} &= 2bR_F - (\omega_m + m_j\frac{N}{k})Ma\\
\frac{dS_N}{dt} &= 2b(S_F+E_F+I_F) - (\omega_m + m_j\frac{N}{k} + (\beta_1+\beta_2)(I_F+I_M+I_N+I_J))S_N + \sigma_1 E_N + \gamma_2 I_N + \omega_2 R_N\\
\frac{dE_N}{dt} &= -(\omega_m + m_j\frac{N}{k} + \sigma_1+\sigma_2+\epsilon)E_N + \beta_1(I_F+I_M+I_N+I_J))S_N + \rho I_N\\
\frac{dI_N}{dt} &= -(\omega_m + m_j\frac{N}{k} + \gamma_1+\gamma_2+\rho)I_N + \beta_2(I_F+I_M+I_N+I_J))S_N + \epsilon E_N\\
\frac{dR_N}{dt} &= -(\omega_m + m_j\frac{N}{k} + \omega)R_N + \sigma_2 E_N + \gamma_2 I_N\\
\frac{dS_J}{dt} &= \omega_m(S_N+Ma) - (\mu + m_j\frac{N}{k} + (\beta_1+\beta_2)(I_F+I_M+I_N+I_J))S_J + \sigma_1 E_J + \gamma_2 I_J + \omega_2 R_J\\
\frac{dE_J}{dt} &= \omega_m E_N - (\mu + m_j\frac{N}{k} + \sigma_1+\sigma_2+\epsilon)E_J + \beta_1(I_F+I_M+I_N+I_J))S_J + \rho I_J\\
\frac{dI_J}{dt} &= \omega_m I_N - (\mu + m_j\frac{N}{k} + \gamma_1+\gamma_2+\rho)I_J + \beta_2(I_F+I_M+I_N+I_J))S_J + \epsilon E_J\\
\frac{dR_J}{dt} &= \omega_m RN - (\mu + m_j\frac{N}{k} + \omega)R_J + \sigma_2 E_J + \gamma_2 I_J
\end{aligned}
$$

2.1 Captive colony likelihood

Likelihoods for the captive colony account for individual, longitudinal data as well as population-level, cross-sectional data. We therefore considered the likelihood that each observed time to seroconversion (e.g., S→I or E for the EIR+ assumption) and seroreversion (e.g., I or E→S for the EIR+ assumption, or the expected immune waning time for the R+ assumption), accounting for 1) the probability of an individual following a certain infection pathway and 2) the probability of a given conversion/reversion time given that pathway. The probabilities of any pathway, including for convenience the probabilities of exiting any cycle between states that are both seronegative or both seropositive, are below. These are the adult transition probabilities, but we use them for the likelihood for all age classes, assuming the chances of following any given path to seroconversion/reversion (and the time taken to do so) is in reality the same for an individual of any age.

$$
\begin{aligned}
Pr(R \to S) &= \frac{\omega}{\omega + m} \\
Pr(E \to S) &= \frac{\sigma_1}{\sigma_1 + \sigma_2 + \epsilon + m} \\
Pr(E \to I) &= \frac{\epsilon}{\sigma_1 + \sigma_2 + \epsilon + m} \\
Pr(E \to R) &= \frac{\sigma_2}{\sigma_1 + \sigma_2 + \epsilon + m} \\
Pr(I \to S) &= \frac{\gamma_1}{\gamma_1 + \gamma_2 + \rho + m} \\
Pr(I \to E) &= \frac{\rho}{\gamma_1 + \gamma_2 + \rho + m} \\
Pr(I \to R) &= \frac{\gamma_2}{\gamma_1 + \gamma_2 + \rho + m} \\
Pr(R \to S) &= \frac{\omega}{\omega + m} \\
Pr(\text{exit I-E}) &= 1 - (\frac{\epsilon}{\sigma_1 + \sigma_2 + \epsilon + m})(\frac{\rho}{\gamma_1 + \gamma_2 + \rho + m}) \\
Pr(\text{exit E-I}) &= Pr(\text{exit I-E}) \\
Pr(\text{exit I-S}) &= 1 - (\frac{\gamma_1}{\gamma_1 + \gamma_2 + \rho + m})(\frac{\beta_2 I^*}{\beta_2 I * + \beta_1 I^* + m}) \\
Pr(\text{exit S-I}) &= Pr(\text{exit I-S}) \\
Pr(\text{exit E-S}) &= 1 - (\frac{\sigma_1}{\sigma_1 + \sigma_2 + \epsilon + m})(\frac{\beta_1 I^*}{\beta_2 I * + \beta_1 I^* + m}) \\
Pr(\text{exit S-E}) &= Pr(\text{exit E-S})
\end{aligned}
$$

Our likelihood function differs based on the serological assumption used (i.e., only immune individuals are seropositive, R+, or all non-susceptible individuals are seropositive, EIR+). Some expected seroconversion/reversion times are the combination of many individual state transition times (e.g., under the R+ assumption, an individual may undergo many cycles of acute and latent infection before seroconverting). The full likelihood function for such times is the convolution of the relevant probability density functions. However, due to computational constraints we made the simplifying assumption of merging these individual state transitions into a single, exponentially-distributed transition with the same expected time.

2.1.1 Assumption: only R seropositive (R+)

Seroreversion probabilities are simple under the assumption that R is the only seropositive compartment. Where t is a single seroreversion time:

$$
\begin{aligned}
&\text{with } \theta = \{\sigma_1, \sigma_2, \epsilon, \rho, \gamma_1, \gamma_2, \omega, m\} \\
&\mathcal{L}(\theta|t) = Pr(R \to S)\omega e^{-\omega t}
\end{aligned}
$$

Seroreversion probabilities under this assumption are somewhat more complicated, because an individual can undergo infinite pathways through the three seronegative states (S, E, and I) by undertaking any number of three different possible cycles (between the S and E states, between S and I, and between I and E). Due to the complexity of this function, we consider only the discrete number of cycles $i \in (0, M)$ for S-E or S-I cycles and $j \in (0, Q)$ for E-I cycles. $M = \text{round}(\frac{\beta_1 I^* \sigma_1}{m(\beta_1 I^* + \sigma_1)})$ in models with S-E cycles and $M = \text{round}(\frac{\beta_2 I^* \gamma_1}{m(\beta_2 I^* + \gamma_1)})$ in models with S-I cycles. No models have both S-E and S-I cycles due to the specification of either β_1 or β_2; $Q = \text{round}(\frac{\rho\epsilon}{m(\rho+\epsilon)})$.

If S-E cycles are possible (i.e., if β_1 is specified), the probability of any seroconversion time t, where $r(\text{path})$ is the expected time to complete a pathway inclusive of any cycles within seronegative states, is:

$$\begin{aligned}
&\text{with } \theta = \{\sigma_1, \sigma_2, \epsilon, \rho, \gamma_1, \gamma_2, \omega, m\} \\
\mathcal{L}(\theta|t) &= Pr(S \to E)Pr(E \to R)\frac{\beta_1 I^* \sigma_2}{\beta_1 I^* + \sigma_2} e^{\frac{\beta_1 I^* \sigma_2}{\beta_1 I^* + \sigma_2} t} \\
&+ \sum_{i=1}^{Q} \sum_{j=0}^{M} (1 - Pr(\text{exit S-E}))^i (1 - Pr(\text{exit E-I}))^j L_{ij} \\
&+ \sum_{j=1}^{M} (1 - Pr(\text{exit E-I}))^j L_j \\
L_i j &= Pr(S \to E)Pr(E \to I)Pr(I \to R) r(S \to E \to I \to R) e^{-r(S \to E \to I \to R)t} \\
L_j &= Pr(S \to E)Pr(E \to I)Pr(I \to R) r(S \to E \to I \to R) e^{-r(S \to E \to I \to R)t} \\
&+ Pr(S \to E)Pr(E \to R) r(S \to E \to R) e^{-r(S \to E \to R)t} \\
&\text{where, given i S-E cycles and j E-I cycles} \\
r(S \to E \to I \to R) &= \frac{1}{j(\frac{1}{\epsilon} + \frac{1}{\rho}) + i(\frac{1}{\sigma_1} + \frac{1}{\beta_1 I^*}) + \frac{1}{\epsilon} + \frac{1}{\gamma_2} + \frac{1}{\beta_1 I^*}} \\
r(S \to E \to R) &= \frac{1}{j(\frac{1}{\epsilon} + \frac{1}{\rho}) + \frac{1}{\sigma_2} + \frac{1}{\beta_1 I^*}}
\end{aligned}$$

If instead β_2 is specified, the probability of any seroconversion time t is:

$$\begin{aligned}
&\text{with } \theta = \{\sigma_1, \sigma_2, \epsilon, \rho, \gamma_1, \gamma_2, \omega, m\} \\
\mathcal{L}(\theta|t) &= Pr(S \to I)Pr(I \to R)\frac{\beta_2 I^* \gamma_2}{\beta_2 I^* + \gamma_2} e^{\frac{\beta_2 I^* \gamma_2}{\beta_2 I^* + \gamma_2} t} \\
&+ \sum_{i=1}^{Q} \sum_{j=0}^{M} (1 - Pr(\text{exit S-I}))^i (1 - Pr(\text{exit I-E}))^j L_{ij} \\
&+ \sum_{j=1}^{M} (1 - Pr(\text{exit I-E}))^j L_j \\
L_i j &= Pr(S \to I)Pr(I \to E)Pr(E \to R) r(S \to I \to E \to R) e^{-r(S \to I \to E \to R)t} \\
L_j &= Pr(S \to I)Pr(I \to E)Pr(E \to R) r(S \to I \to E \to R) e^{-r(S \to I \to E \to R)t} \\
&+ Pr(S \to I)Pr(I \to R) r(S \to I \to R) e^{-r(S \to I \to R)t} \\
&\text{where, given i S-I cycles and j I-E cycles} \\
r(S \to I \to E \to R) &= \frac{1}{j(\frac{1}{\epsilon} + \frac{1}{\rho}) + i(\frac{1}{\gamma_1} + \frac{1}{\beta_2 I^*}) + \frac{1}{\rho} + \frac{1}{\sigma_2} + \frac{1}{\beta_2 I^*}} \\
r(S \to I \to R) &= \frac{1}{j(\frac{1}{\epsilon} + \frac{1}{\rho}) + \frac{1}{\gamma_2} + \frac{1}{\beta_2 I^*}}
\end{aligned}$$

2.1.2 Assumption: E and R seropositive (ER+)

Seroreversion under the ER+ assumption includes both R to S transitions and E to I or S transitions. The probability of any seroreversion time t given either specification of β is:

$$\begin{aligned}
&\text{with } \theta = \{\sigma_1, \sigma_2, \epsilon, \rho, \gamma_1, \gamma_2, \omega, m\} \\
&\mathcal{L}(\theta|t) = \frac{Pr(S \to E)S^* + Pr(I \to E)I^*}{Pr(S \to E)S^* + Pr(I \to E)I^* + Pr(I \to R)I^*}(\\
&\quad Pr(E \to R)Pr(R \to S)(\frac{\sigma_2\omega}{\sigma_2 + \omega})e^{-\frac{\sigma_2\omega}{\sigma_2+\omega}t} \\
&\quad + Pr(E \to I)\epsilon e^{-\epsilon t} \\
&\quad + Pr(E \to S)\gamma_1 e^{-\gamma_1 t}) \\
&\quad + \frac{Pr(I \to R)I^*}{Pr(S \to E)S^* + Pr(I \to E)I^* + Pr(I \to R)I^*} Pr(R \to S)\omega e^{-\omega t}
\end{aligned}$$

Seroconversion under the ER+ assumption includes both S to E transitions and I to E or R transitions and may include cycles between the seronegative states S and I:

$$\begin{aligned}
&\text{with } \theta = \{\sigma_1, \sigma_2, \epsilon, \rho, \gamma_1, \gamma_2, \omega, m\} \\
&\mathcal{L}(\theta|t) = \frac{Pr(R \to S)R^* + Pr(E \to S)E^*}{Pr(R \to S)R^* + Pr(E \to S)E^* + Pr(E \to I)E^*}(\\
&\quad Pr(S \to I)Pr(I \to R)(\frac{\beta_2 I^*\gamma_2}{\beta_2 I^* + \gamma_2})e^{-\frac{\beta_2 I^*\gamma_2}{\beta_2 I^*+\gamma_2}t} \\
&\quad + Pr(S \to E)\beta_1 I^* e^{-\beta_1 I^* t} \\
&\quad + Pr(S \to I)Pr(I \to E)(\frac{\beta_2 I^*\rho}{\beta_2 I^* + \rho})e^{-\frac{\beta_2 I^*\rho}{\beta_2 I^*+\rho}t} \\
&\quad + \sum_{i=1}^{M}(1 - Pr(\text{exit I-S}))^i L_i)) \\
&\quad + \frac{Pr(E \to I)E^*}{Pr(R \to S)R^* + Pr(E \to S)E^* + Pr(E \to I)E^*}(\\
&\quad Pr(I \to R)\gamma_2 e^{-\gamma_2 t} \\
&\quad + Pr(I \to E)\rho e^{-\rho t} \\
&\quad + \sum_{j=1}^{Q}(1 - Pr(\text{exit I-S}))^j L_j) \\
&L_i = Pr(S \to I)Pr(I \to E)r(S \to I \to E)e^{-r(S\to I\to E)t} \\
&\quad \text{where, given i cycles} \\
&r(S \to I \to E) = \frac{\gamma_1(\beta_2 I^*)^2\rho}{\gamma_1(\beta_2 I^*) + \gamma_1\beta_2 I^*\rho + \beta_2 I^*\rho i(\gamma_1 + \beta_2 I^*)} \\
&L_j = Pr(I \to E)r(I \to E)e^{-r(I\to E)t} \\
&\quad \text{where, given j I-E cycles} \\
&r(I \to E) = \frac{\gamma_1\beta_2 I^*\rho}{\rho j(\gamma_1 + \beta_2 I^*) + \gamma_1\beta_2 I^*}
\end{aligned}$$

2.1.3 Assumption: E, I, and R seropositive (EIR+)

In a model with transmission from S to E, the likelihood of a seroreversion time t is:

$$\begin{aligned}
&\text{with } \theta = \{\sigma_1, \sigma_2, \epsilon, \rho, \gamma_1, \gamma_2, \omega, m\}\\
\mathcal{L}(\theta|t) &= Pr(E \to S)\sigma_1 e^{-\sigma_1 t}\\
&+ Pr(E \to R)Pr(R \to S)(\frac{\sigma_2\omega}{\sigma_2+\omega})e^{-\frac{\sigma_2\omega}{\sigma_2+\omega}t}\\
&+ Pr(E \to I)Pr(I \to S)(\frac{\epsilon\gamma_1}{\epsilon+\gamma_1})e^{-\frac{\epsilon\gamma_1}{\epsilon+\gamma_1}t}\\
&+ Pr(E \to I)Pr(I \to R)Pr(R \to S)(\frac{\epsilon\gamma_2\omega}{\epsilon\omega+\epsilon\gamma_2+\gamma_2\omega})e^{-\frac{\epsilon\gamma_2\omega}{\epsilon\omega+\epsilon\gamma_2+\gamma_2\omega}t}\\
&+ \sum_{i=1}^{M}(1-Pr(\text{exit}))^i L_i\\
L_i &= Pr(E \to S)r(E \to S)e^{-r(E\to S)t}\\
&+ Pr(E \to R)Pr(R \to S)r(E \to R \to S)e^{-r(E\to R\to S)t}\\
&+ Pr(E \to I)Pr(I \to R)Pr(R \to S)r(E \to I \to R \to S)e^{-r(E\to I\to R\to S)t}\\
&\text{where, given i cycles}\\
r(E \to S) &= \frac{\epsilon\rho\sigma_1}{\sigma_1 i(\epsilon+\rho)+\rho\epsilon}\\
r(E \to R \to S) &= \frac{\sigma_2\omega\rho\epsilon}{\epsilon\rho\omega+\epsilon\rho\sigma_2+\sigma_2\omega i(\epsilon+\rho)}\\
r(E \to I \to R \to S) &= \frac{\rho\epsilon\gamma_2\omega}{\omega\gamma_2 i(\rho+\epsilon)+\gamma_2\omega\rho+\epsilon\omega\rho+\gamma_2\rho\epsilon}
\end{aligned}$$

In a model with transmission from S to I, the likelihood of a seroreversion time t is:

$$
\begin{aligned}
\mathcal{L}(\theta|t) &= Pr(I \to S)\gamma_1 e^{-\gamma_1 t} \\
&+ Pr(I \to R)Pr(R \to S)(\frac{\gamma_2\omega}{\gamma_2+\omega})e^{-\frac{\gamma_2\omega}{\gamma_2+\omega}t} \\
&+ Pr(I \to E)Pr(E \to S)(\frac{\rho\sigma_1}{\rho+\sigma_1})e^{-\frac{\rho\sigma_1}{\rho+\sigma_1}t} \\
&+ Pr(I \to E)Pr(E \to R)Pr(R \to S)(\frac{\rho\sigma_2\omega}{\rho\omega+\rho\sigma_2+\sigma_2\omega})e^{-\frac{\rho\sigma_2\omega}{\rho\omega+\rho\sigma_2+\sigma_2\omega}t} \\
&+ \sum_{i=1}^{M}(1-Pr(\text{exit I-E}))^i L_i \\
L_i &= Pr(I \to S)r(I \to S)e^{-r(I\to S)t} \\
&+ Pr(I \to R)Pr(R \to S)r(I \to R \to S)e^{-r(I\to R\to S)t} \\
&+ Pr(I \to E)Pr(E \to S)r(I \to E \to S)e^{-r(I\to E\to S)t} \\
&+ Pr(I \to E)Pr(E \to R)Pr(R \to S)r(I \to E \to R \to S)e^{-r(I\to E\to R\to S)t} \\
&\text{where, given i cycles} \\
r(I \to S) &= \frac{\epsilon\rho\gamma_1}{\gamma_1 i(\epsilon+\rho)+\rho\epsilon} \\
r(I \to R \to S) &= \frac{\gamma_2\omega\rho\epsilon}{\epsilon\rho\omega+\epsilon\rho\gamma_2+\gamma_2\omega i(\epsilon+\rho)} \\
r(I \to E \to S) &= \frac{\rho\epsilon\sigma_1}{\sigma_1 i(\rho+\epsilon)+\sigma_1\epsilon+\rho\epsilon} \\
r(I \to E \to R \to S) &= \frac{\rho\epsilon\sigma_2\omega}{\omega\sigma_2 i(\rho+\epsilon)+\sigma_2\omega\rho+\epsilon\omega\rho+\sigma_2\rho\epsilon}
\end{aligned}
$$

For both models with S to E and S to I transmission, the likelihood associated with a seroconversion time t is simply the expected transmission time. However because measurements of captive colony serology were collected at inconsistent intervals, on two occasions more than a year apart, we assigned each seroconversion time and seroreversion time a minimum and maximum value (based on the earliest and latest times in the preceding and following sampling periods) and assumed a uniform distribution between the two. The true likelihood of a seroconversion/seroreversion time t with a maximum time t_{max} and minimum time t_{min}, if the calculated seroconversion/seroreversion rate is r, is actually

$$re^{-rt} = e^{-rt_{min}} - e^{-rt_{max}}(\frac{1}{t_{max}-t_{min}})$$

. For example, the full likelihood of a seroconversion time under the EIR+ assumption is:

$$
\begin{aligned}
\mathcal{L}(\theta|t) &= \frac{\beta\bar{I}}{\beta\bar{I}+m}\beta\bar{I}e^{-\beta\bar{I}t} \\
&\text{where } \bar{I} \text{ is the mean number of infecteds over the past period } t \\
\mathcal{L}(\theta|T \sim U(t_{min}, t_{max})) &= \int_{t_{min}}^{t_{max}} Pr(t \sim T)\frac{\beta\bar{I}}{\beta\bar{I}+m}\beta\bar{I}e^{-\beta\bar{I}t}dt \\
\mathcal{L}(\theta|T \sim U(t_{min}, t_{max})) &= \frac{\beta\bar{I}}{\beta\bar{I}+m}(e^{-\beta\bar{I}t_{min}} - e^{-\beta\bar{I}t_{max}})
\end{aligned}
$$

Finally, the likelihood function includes a term for measured seroprevalences over time. This is the same as the seroprevalence likelihood for the cross-sectional data on wild-caught bats, but for multiple time points

at one location and with all age classes pooled due to incomplete age information for the captive bats. Where P_x is the simulated seroprevalence at sampling timepoint x; n_x and p_x are the observed numbers of all bats and seropositive bats at time x, respectively; and $\bar{T}_R$ and $\bar{T}_C$ are the sets of all observed seroreversion times and seroconversion times, respectively, the final function is:

$$\mathcal{L}(\theta|\bar{T}_R, \bar{T}_C, n, p) = \mathcal{L}(\theta|\bar{T}_R)) * \mathcal{L}(\theta|\bar{T}_C)) * \prod_{x=1}^{22} \binom{n_x}{p_x} P_x^{p_x}(1-P_x)^{n_x-p_x}$$

2.2 Captive colony fitting

We fit the captive colony likelihood in two steps:

1. Fitting the deterministic variant of the model by maximising the product of the seroconversion and seroreversion likelihoods described in Section 2.1. We selected initial parameter values based on a random Latin hypercube sample ($n = 100$) and optimised each using the Nelder-Mead algorithm as implemented by `optim()` in R [7]. For both initial parameter values and throughout optimisation, we constrained parameters within the bounds listed in Table 1 in the main text.

2. With starting parameters set to those returned by step 1, fit the stochastic variant of the model (simulated via adaptive tau-leaping, see Section 2.3) via particle filtering as outlined below.

Particle filtering algorithm. We followed a similar algorithm to [3], although instead of iterating across time points and taking the likelihoods of observed cross-sections, we iterated across individual seroconversion and seroreversion times and took the likelihood of each. We also fixed the initial state sizes (at 5 each initial infected adult males and adult females, with other compartments fixed to their values after 300 years of the best-fitting deterministic model) and allowed the simulations to run for 8 years to assess persistence; we assumed the final year represented equilibrium and calculated seroconversion likelihoods based on the mean number of infecteds across the final year of simulation. The standard deviation of the random walk for each parameter was set to 50% of its initial estimate, with a cooling factor of 1% per iteration.

We calculated AIC independently for each particle. We calculated a vector of model weights (W) for any one assumptions set as the mean of weights across 1000 quantiles:

$$W = \frac{1}{1000} \sum_{i=1}^{1000} w(\Delta_{[i/1000]}),$$

where Δ for any model represents ΔAIC, w is the vector of Akaike weights for all models m (with $M = 46$ models) $w_m = \frac{e^{-\Delta_m/2}}{\sum_n^M e^{-\Delta_n/2}}$, and $i/1000$ is the relevant quantile.

2.3 Stochastic simulation algorithm

We implemented adaptive tau-leaping [4] using the Rcpp package [1]. We set maximum and minimum time steps (10^{-3} days $\leq \tau \leq 0.5$ days) for tau leaping. By default, time is incremented by the maximum $\tau = 0.5$ days unless there are fewer than 10 individuals in any compartment; in these cases, τ is set to the $\min(\frac{1}{\{r_-\}})$ where $\{r_-\}$ is the set of rates causing depletion in the compartment.

Once a time step τ is chosen, events occur based on random events $\sim Pois(\{r\}\tau)$. If these events cause any compartment to go negative, τ is repeatedly halved until it falls below the minimum threshold, at which point we took a single step with the Gillespie stochastic simulation algorithm.

2.4 Feature definitions

Various parameters and combinations of parameters create mechanistic features in our submodels. We refer to the following parametric definitions in the main text, where OR is an inclusive or (i.e., at least one of the conditions is met):

- Recurring infection: $\rho > 0$ AND $\epsilon > 0$
- Lifelong immunity: ($\gamma_2 > 0$ OR $\sigma_2 > 0$) AND $\omega = 0$
- Temporary immunity: ($\gamma_2 > 0$ OR $\sigma_2 > 0$) AND $\omega > 0$
- No immunity: $\gamma_2 > 0$ AND $\sigma_2 > 0$
- Clearance from I: $\gamma_1 > 0$ OR $\gamma_2 > 0$
- Clearance from E: $\sigma_1 > 0$ OR $\sigma_2 > 0$
- Reinfection: $\sigma_1 > 0$ OR $\gamma_1 > 0$ OR (($\gamma_2 > 0$ OR $\sigma_2 > 0$) AND $\omega > 0$)
- Non-infectious infections: $\beta_1 > 0$ AND ($\sigma_1 > 0$ OR ($\sigma_2 > 0$ AND $\omega > 0$))

3 Supplementary results

References

[1] Dirk Eddelbuettel and James Joseph Balamuta. Extending R with C++: A Brief Introduction to Rcpp. *PeerJ Preprints*, 5:e3188v1, aug 2017.

[2] D T Hayman, R McCrea, O Restif, R Suu-Ire, A R Fooks, J L Wood, A A Cunningham, and J M Rowcliffe. Demography of straw-colored fruit bats in Ghana. *J Mammal*, 93(5):1393–1404, 2012.

[3] E L Ionides, C Bretó, and A A King. Inference for nonlinear dynamical systems. *Proceedings of the National Academy of Sciences of the United States of America*, 103(49):18438–18443, 2006.

[4] A J Peel, J R C Pulliam, A D Luis, R K Plowright, T J O Shea, D T S Hayman, J L N Wood, C T Webb, O Restif, and Proc R Soc B. The effect of seasonal birth pulses on pathogen persistence in wild mammal populations The effect of seasonal birth pulses on pathogen persistence in wild mammal populations. *Proceedings of The Royal Society Biological Sciences*, 281:1–9, 2014.

[5] Alison J. Peel, Kate S. Baker, David T. S. Hayman, Christopher C. Broder, Andrew A. Cunningham, Anthony R. Fooks, Romain Garnier, James L. N. Wood, and Olivier Restif. Support for viral persistence in bats from age-specific serology and models of maternal immunity. *Scientific Reports*, 8(1):3859, 2018.

[6] Alison J. Peel, James L. N. Wood, Kate S. Baker, Andrew C. Breed, Arlindo De Carvalho, Andrés Fernández-Loras, Harrison Sadiki Gabrieli, Guy-Crispin Gembu, Victor A. Kakengi, Potiphar M. Kaliba, Robert M. Kityo, Tiziana Lembo, Fidel Esono Mba, Daniel Ramos, Iñaki Rodriguez-Prieto, Richard Suu-Ire, Andrew A. Cunningham, and David T. S. Hayman. How Does Africa's Most Hunted Bat Vary Across the Continent? Population Traits of the Straw-Coloured Fruit Bat (<i>Eidolon helvu</i> m) and Its Interactions with Humans. *Acta Chiropterologica*, 19(1):77–92, 2017.

[7] R Core Team. *R: A Language and Environment for Statistical Computing*. R Foundation for Statistical Computing, Vienna, Austria, 2018.

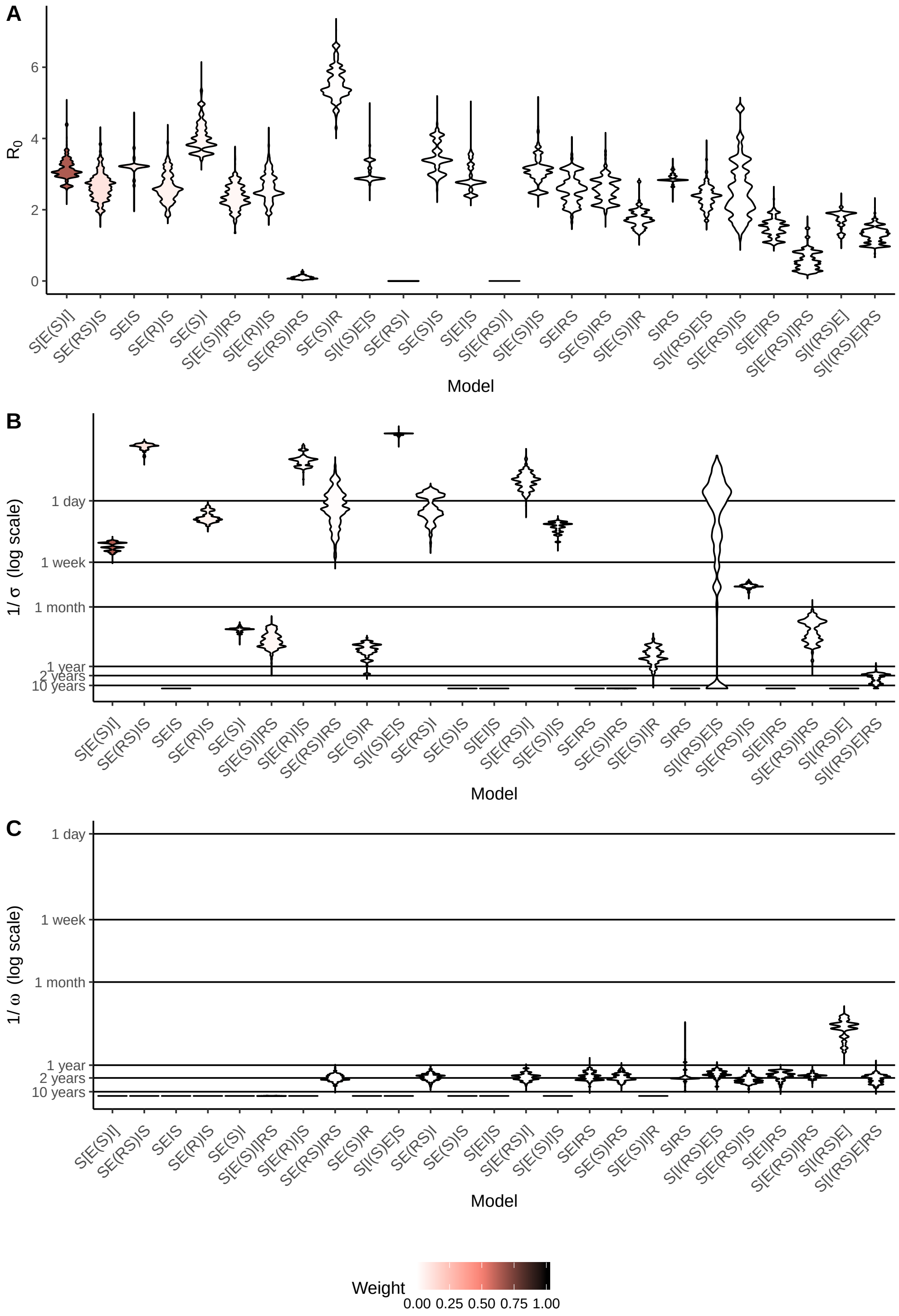

Figure 2: Distributions of predicted parameter values under the EIR+ serological assumption. R_0 values (A), time to clearance from E ($1/\sigma$, B), and immune waning durations ($1/\omega$, C) are weighted by particle likelihood in last ten iterations of stochastic captive colony fitting procedure. Models are ordered according to decreasing weight.

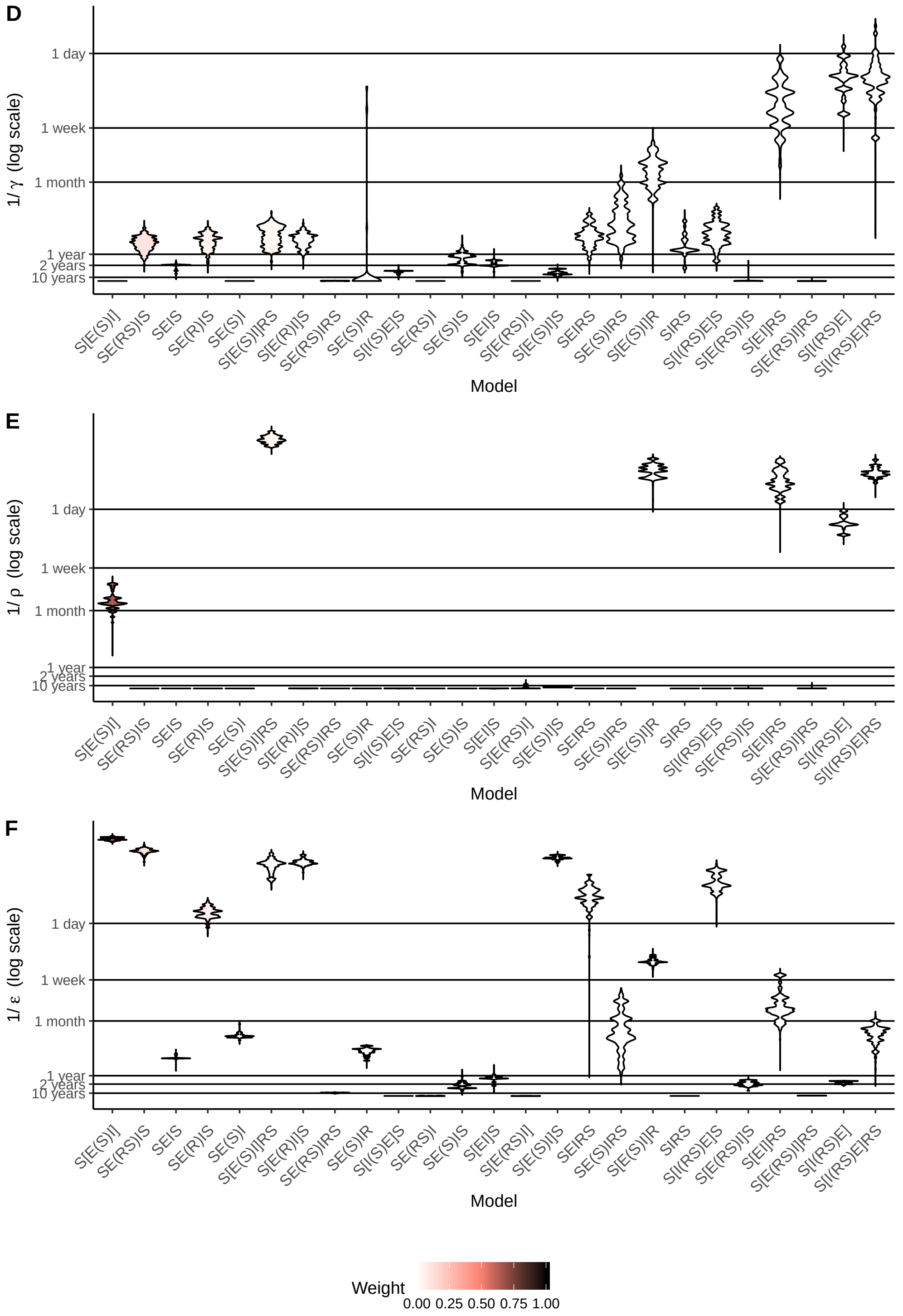

Figure 3: Distributions of predicted parameter values under the EIR+ serological assumption (continued). Time to clearance from I ($1/\sigma$, D), incubation time or time to recurrence ($1/\rho$, E), and time to revert to latency ($1/\epsilon$, F) are weighted by particle likelihood in last ten iterations of stochastic captive colony fitting procedure. Models are ordered according to decreasing weight.

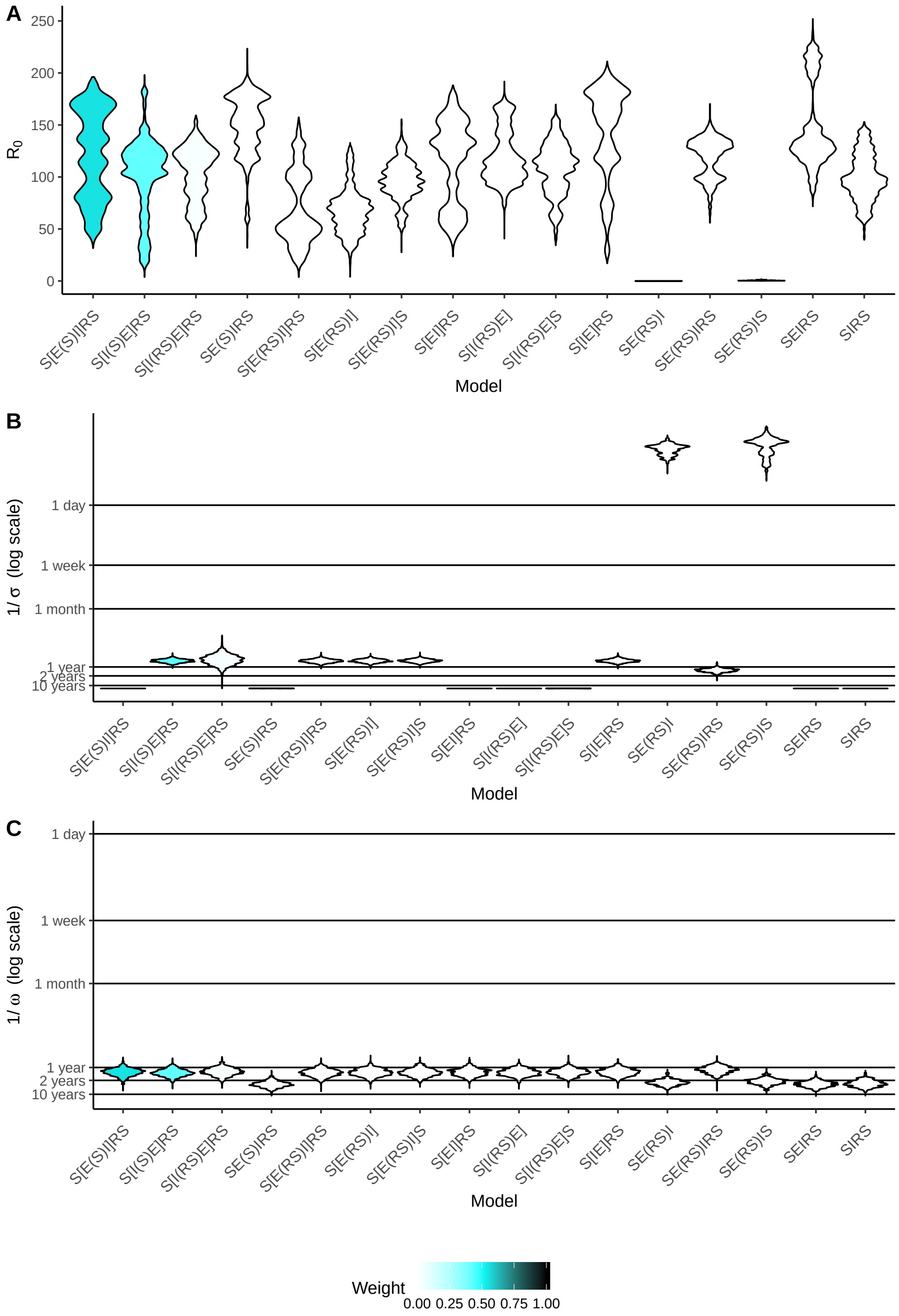

Figure 4: Distributions of predicted parameter values under the R+ serological assumption. R_0 values (A), time to clearance from E ($1/\sigma$, B), and immune waning durations ($1/\omega$, C) are weighted by particle likelihood in last ten iterations of stochastic captive colony fitting procedure. Models are ordered according to decreasing weight.

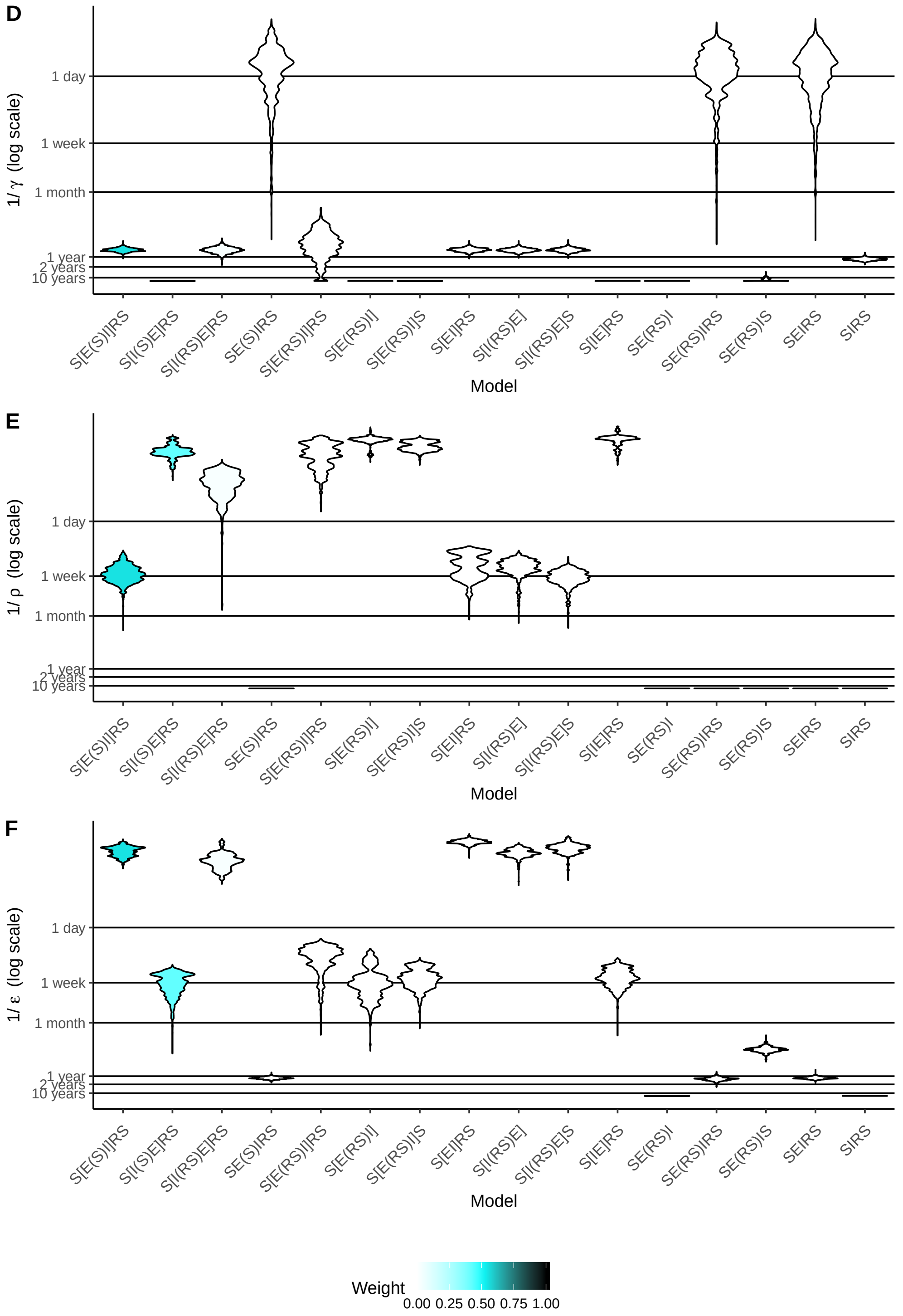

Figure 5: Distributions of predicted parameter values under the R+ serological assumption (continued). Time to clearance from I ($1/\sigma$, D), incubation time or time to recurrence ($1/\rho$, E), and time to revert to latency ($1/\epsilon$, F) are weighted by particle likelihood in last ten iterations of stochastic captive colony fitting procedure. Models are ordered according to decreasing weight.

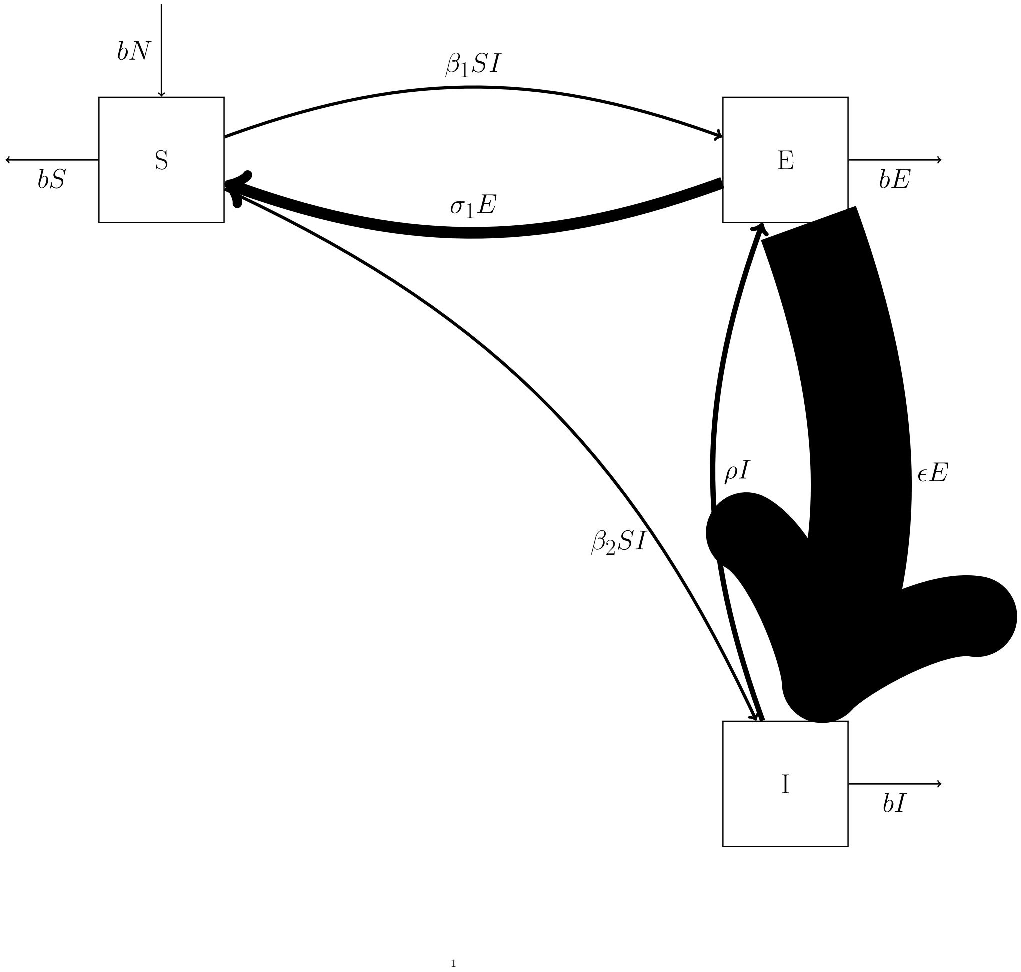

Figure 6: Flow diagram of top model (S[E(S)I]) under EIR+ assumption, with line weights corresponding to the square root of fit parameter estimates.

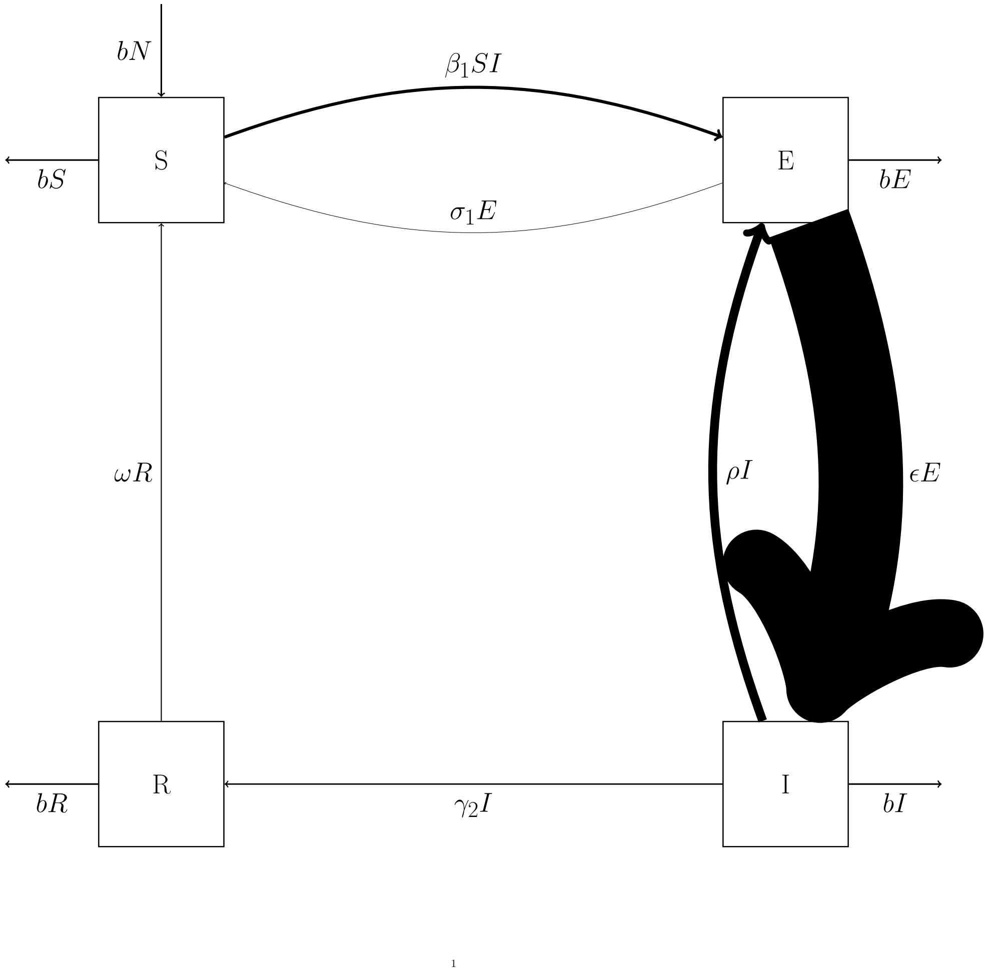

Figure 7: Flow diagram of top model (S[E(S)I]RS) under R+ assumption, with line weights corresponding to the square root of fit parameter estimates.
